# Prioritized Expression of *BTN2* of *Saccharomyces cerevisiae* under Pronounced Translation Repression Induced by Severe Ethanol Stress

**DOI:** 10.3389/fmicb.2016.01319

**Published:** 2016-08-23

**Authors:** Yukina Yamauchi, Shingo Izawa

**Affiliations:** Laboratory of Microbial Technology, Department of Applied Biology, Graduate School of Science and Technology, Kyoto Institute of TechnologyKyoto, Japan

**Keywords:** *Saccharomyces cerevisiae*, ethanol stress, translation repression, alcoholic fermentation, preferential translation, *BTN2*

## Abstract

Severe ethanol stress (>9% ethanol, v/v) as well as glucose deprivation rapidly induces a pronounced repression of overall protein synthesis in budding yeast *Saccharomyces cerevisiae.* Therefore, transcriptional activation in yeast cells under severe ethanol stress does not always indicate the production of expected protein levels. Messenger RNAs of genes containing heat shock elements can be intensively translated under glucose deprivation, suggesting that some mRNAs are preferentially translated even under severe ethanol stress. In the present study, we tried to identify the mRNA that can be preferentially translated under severe ethanol stress. *BTN2* encodes a v-SNARE binding protein, and its null mutant shows hypersensitivity to ethanol. We found that *BTN2* mRNA was efficiently translated under severe ethanol stress but not under mild ethanol stress. Moreover, the increased Btn2 protein levels caused by severe ethanol stress were smoothly decreased with the elimination of ethanol stress. These findings suggested that severe ethanol stress extensively induced *BTN2* expression. Further, the *BTN2* promoter induced protein synthesis of non-native genes such as *CUR1*, *GIC2*, and *YUR1* in the presence of high ethanol concentrations, indicating that this promoter overcame severe ethanol stress-induced translation repression. Thus, our findings provide an important clue about yeast response to severe ethanol stress and suggest that the *BTN2* promoter can be used to improve the efficiency of ethanol production and stress tolerance of yeast cells by modifying gene expression in the presence of high ethanol concentration.

## Introduction

Budding yeast *Saccharomyces cerevisiae* produces ethanol through alcoholic fermentation. Ethanol concentrations in wine must and Japanese *sake* mash reach high levels in the final stage of brewing. High ethanol concentration exerts adverse effects on yeast cells and inhibits yeast cell growth and viability by inducing severe stress. Ethanol concentration of >9% (v/v) blocks the nuclear export of bulk poly(A)^+^ mRNA and represses translation initiation in yeast cells (Takemura et al., 2004; Izawa et al., 2005a,b; Iwaki et al., 2013; Yamamoto and Izawa, 2013). Repression of overall protein synthesis in yeast cells under severe ethanol stress indicates that increased mRNA expression does not always result in the expected increase in protein expression (Izawa, 2010, 2015). Pronounced repression of overall protein synthesis seems to be one of the primary causes of growth suppression of yeast cells under severe ethanol stress.

During translation repression, untranslated mRNAs leave the translation apparatus and form the cytoplasmic messenger ribonucleoprotein (mRNP) granules such as processing bodies (P-bodies) and stress granules (SGs) under severe stress conditions. It has been reported that glucose deprivation, NaN_3_, high vanillin concentration, and robust heat shock repress translation activity in yeast cells and induce the formation of P-bodies and SGs (Teixeira et al., 2005; Balagopal and Parker, 2009; Buchan and Parker, 2009; Grousl et al., 2009; Buchan et al., 2011; Nguyen et al., 2014, 2015). P-bodies and SGs play important roles in the regulation of gene expression under severe stress (Balagopal and Parker, 2009; Buchan and Parker, 2009). Severe ethanol stress also activates the formation of P-bodies and SGs in yeast cells (Izawa et al., 2007; Kato et al., 2011).

Proteins required for stress tolerance are intensively synthesized under severe stress despite the pronounced repression of translation activity. Glucose deprivation rapidly causes a reduction in overall protein synthesis in yeast cells (Ashe et al., 2000). Zid and O’Shea (2014) reported that mRNAs of genes encoding small heat shock proteins (sHSPs), such as *HSP26* and *HSP30*, are preferentially translated during glucose deprivation. Promoter sequences of these genes contain heat shock elements (HSEs) that not only affect the mRNA levels of these genes but also affect the efficiency of mRNA translation during glucose deprivation. Recently, we also reported that the *BDH2* promoter and *ADH7* promoter*-*driven mRNAs were preferentially translated under severe vanillin stress, which induces translation repression (Nguyen et al., 2015; Ishida et al., 2016). However, no information is available on yeast mRNAs that are efficiently translated under severe ethanol stress. Identification of these mRNAs is important for understanding the response of yeast cells to severe stress.

Therefore, we examined the expression of previously reported genes associated with ethanol tolerance. In the present study, we focused on *BTN2* whose deficiency induces hypersensitivity to ethanol (Espinazo-Romeu et al., 2008; Yang et al., 2011). *BTN2* encodes a v-SNARE binding protein that is involved in intracellular protein trafficking (Kama et al., 2007) and plays a role in protein deposition in the nucleus (Miller et al., 2015). Because Btn2 is important for the correct localization of various proteins, *btn2*Δ cells show pleiotropic phenotypes, including decreased resistance to ethanol, acidic pH, hydrostatic pressure, and L-canavanine (Chattopadhyay et al., 2000; Chattopadhyay and Pearce, 2002; Kim et al., 2005; Espinazo-Romeu et al., 2008; Yang et al., 2011). We found that *BTN2* mRNA was efficiently translated under severe ethanol stress and that Btn2 protein levels decreased after ethanol elimination. Moreover, the *BTN2* promoter induced the expression of non-native genes such as *CUR1*, *GIC2*, and *YUR1* under severe ethanol stress. These findings suggested that *BTN2* expression responded to severe ethanol stress and that the *BTN2* promoter could be used to improve ethanol tolerance or produce useful proteins during brewing by modifying yeast gene expression under severe ethanol stress.

## Materials and Methods

### Strains and Medium

*Saccharomyces cerevisiae* strain BY4742 (*MATα his3*Δ*1 ura3*Δ*0 leu2*Δ*0 lys2*Δ*0*) was used in this study. Yeast cells were cultured in SD medium (2% glucose, 0.67% yeast nitrogen base without amino acids, 20 mg/L uracil, 30 mg/L L-lysine HCl, 100 mg/L L-leucine, and 20 mg/L L-histidine HCl) at 28°C with reciprocal shaking (120 rpm).

### Chemicals and Analysis Methods

Exponentially growing yeast cells were harvested when OD_600_ of the culture medium reached 0.5 and were treated with various concentrations of ethanol (Wako, Osaka, Japan). Cycloheximide (CHX) was purchased from Nacalai Tesque, Kyoto, Japan. Polysome profile analysis was performed using a method described by Inada and Aiba (2005). The preparation of yeast extract and sucrose gradient separation for polysome profile analysis was performed using a gradient master and fractionator (107–201 M and 152–002, BioComp Instruments, Fredericton, NB, Canada). Polysome ratio was determined as the percentage of area under polysomal ribosome peaks relative to that under total ribosome peaks, according to a method described by Hofmann et al. (2012). Fluorescence microscopic analysis was performed using a Leica AF6500 fluorescence microscope system (Leica Microsystems Vertrieb GmbH, Hessen, Germany).

### Plasmids

Sequences of primers used for constructing plasmids are listed in **Table 1**. Genomic DNA from *S. cerevisiae* strain BY4742 was used as the template for amplifying yeast genes by PCR.

**Table 1:** List of primers used in plasmid construction.

Name	Sequence
*BTN2*_Orf_*-*F	5′-ACTTTTCTAGATTGGTTTAGTTAAGCATGA-3′
*BTN2_Orf_-*R	5′-TATCTCTCGAGATATCTCCTCAATAATAGA-3′
*BTN2*_FLAG-Ter_-F	5′-GATATCTCGAGTCGACTACAAGGATGACGATGACAAGTAATGGGTGATAATACATACTCCCCATC-3′
*BTN2*_FLAG-Ter_-R	5′-ATAATGGTACCAAAATCACGGATACTAATA-3′
*BTN2*_Pro_*2-*F	5′-GGGGTACTAGTGCCTATAAGTTCGAAGCCA-3′
*BTN2*_Pro_*2-*R	5′-GAAAAGAATTCTATATTGTAATGGGGTCTA-3′
*CUR1-*F	5′-AACCAAAAGAAAATAACTAATAGACCCCATTACAATATAGAAATGGCTGCCGCATGCATTTGTCAACCTAATCTT-3′
*CUR1-*R	5′-TTATCACCCATTACTTGTCATCGTCATCCTTGTAGTCCTCGAGCCGCCCATTCAATCTTCTAGATACTTCCTT-3′
*GIC2-*F	5′-AACCAAAAGAAAATAACTAATAGACCCCATTACAATATAGAAATGACTAGTGCAAGTATTACCAATACTGGAAAC-3′
*GIC2-*R	5′-TTATCACCCATTACTTGTCATCGTCATCCTTGTAGTCCTCGAGAGTTTGCAGGGGCTCGAGCTGGTTGAAAGA-3′
*YUR1-*F	5′-AACC AAAAGAAAATAACTAATAGACCCCATTACAATATAGAAATGGCAAAAGGAGGCTCGCTATACATCGTTGGC-3′
*YUR1-*R	5′-TTATCACCCATTACTTGTCATCGTCATCCTTGTAGTCCTCGAGAATCTCGTCTTGCTCTTCTTTTAAGAAATATTTGCCG-3′
*ADH6_FLAG-Ter_-*F	5′-GCAAGCTCGAGCTGACTACAAGGATGACGATGACAAGTAGGTTGTCAAGCTCTTGATAAATG-3′
ADH6_FLAG-Ter_-R	5′-GAAAAGGTACCCAGATCTACCACCAAACCT-3′
*TEF1*_FLAG-Ter_*-*F	5′-GCAAGCTCGAGCTGACTACAAGGATGACGATGACAAGTAAGGAGATTGATAAGACTTTTCTAG-3′
TEF1_FLAG-Ter_-R	5′-CGTAAAACTAGATAGCAGTTTGGTACCTAT-3′


#### YIp*-BTN2-FLAG-BTN2*_Ter_

The integrate-type plasmid YIp-*BTN2-FLAG* was constructed to determine Btn2 protein expression. This plasmid contained a part of the *BTN2* open reading frame (ORF), a FLAG tag sequence (encoded by 24 nt) immediately upstream of the stop codon, and a 3′-flanking region of *BTN2*. A 0.6-kbp fragment encoding the part of *BTN2*_Orf_ and a 0.2-kbp fragment encoding *BTN2*_FLAG-Ter_ were amplified using primer sets *BTN2*_Orf_ - F/*BTN2*_Orf_ - R and *BTN2*_FLAG_*_-_*_Ter_-F/*BTN2*_FLAG_*_-_*_Ter_-R, respectively. Amplicons obtained were digested using *Xba*I/*Xho*I and *Xho*I/*Kpn*I, respectively, and were cloned into the *Xba*I/*Kpn*I sites of pJK67 (Kahana et al., 1998) to construct YIp-*BTN2-FLAG-BTN2*_Ter_. To integrate the *BTN2-FLAG-BTN2*_Ter_ gene in the chromosomal *BTN2* locus, YIp-*BTN2-FLAG-BTN2*_Ter_ was linearized by digesting it with *Hpa*I and was introduced into yeast cells.

#### YIp*-BTN2-FLAG-ADH6*_Ter_ and YIp*-BTN2-FLAG-TEF1*_Ter_

A 0.4-kbp fragment encoding *ADH6*_FLAG-Ter_ and a 0.7-kbp fragment encoding *TEF1*_FLAG-Ter_ were amplified using primer sets *ADH6*_FLAG_*_-_*_Ter_-F/*ADH6*_FLAG_*_-_*_Ter_-R and *TEF1*_FLAG_*_-_*_Ter_-F/*TEF1*_FLAG_*_-_*_Ter_-R, respectively. Amplicons obtained were digested using *Xho*I/*Kpn*I and were cloned into the *Xho*I/*Kpn*I sites of YIp-*BTN2-FLAG-BTN2*_Ter_ to construct YIp*-BTN2-FLAG-ADH6*_Ter_ and YIp*-BTN2-FLAG-TEF1*_Ter_, respectively. To integrate them in the chromosomal *BTN2* locus, they were linearized with *Hpa*I and introduced into yeast cells.

#### pRS316-*BTN2*_Pro/FLAG-Ter_

A 1.2 kbp fragment of the *BTN2* promoter region (*BTN2*_Pro_) and a 0.2 kbp *BTN2*_FLAG-Ter_ fragment were amplified using primer sets *BTN2*_Pro_2-F/*BTN2*_Pro_2-R and *BTN2*_FLAG_*_-_*_Ter_-F/*BTN2*_FLAG_*_-_*_Ter_-R, respectively, and were cloned into the *Spe*I/*Eco*RI sites and the *Xho*I/*Kpn*I sites, respectively, of pRS316 (Sikorski and Hieter, 1989) to construct pRS316-*BTN2*_Pro/FLAG-Ter_, that was named pYY2712. ORFs of *CUR1* (0.8 kbp), *GIC2* (1.2 kbp), and *YUR1* (1.3 kbp) were amplified using primer sets *CUR1*-F/*CUR1*-R, *GIC2*-F/*GIC2*-R, and *YUR1*-F/*YUR1*-R, respectively. Amplicons obtained were cloned into the pYY2712 that was previously digested with *Eco*RI/*Sal*I to construct pYY plasmid series, pYY-*CUR1-FLAG* (pRS316-*BTN2*_Pro_-*CUR1*_Orf_-*BTN2*_FLAG-Ter_), pYY-*GIC2-FLAG* (pRS316-*BTN2*_Pro_-*GIC2*_Orf_-*BTN2*_FLAG-Ter_), and pYY-*YUR1-FLAG* (pRS316-*BTN2*_Pro_-*YUR1*_Orf_-*BTN2*_FLAG-Ter_), respectively, by using gap repair cloning method (Goto and Nagano, 2013).

### Quantitative Reverse Transcription-PCR

Relative mRNA levels of the *BTN2*, *HSP30*, *CUR1-FLAG*, *GIC2-FLAG*, and *YUR1-FLAG* genes were determined by performing quantitative reverse transcription-PCR (qRT-PCR). Total RNA was extracted from yeast cells by using a method described by Schmitt et al. (1990). RNA obtained was reverse transcribed to cDNA by using ReverTra Ace qPCR RT Master Mix FSQ-201 (Toyobo, Osaka, Japan), according to the manufacturer’s instructions. Quantitative PCR was performed using Thermal Cycler Dice Real Time System Lite (Takara Bio Inc., Shiga, Japan) and SYBR^®^ Premix Ex Taq^TM^ II (Takara Bio Inc., Shiga, Japan). Comparison of mRNA expression levels was performed by normalizing the mRNA level of each gene to that of *ACT1* (Takahashi et al., 2011). The mRNA level was expressed as the ratio of normalized mRNA level of the target gene to that of the reference gene. Oligonucleotide sequences of primers used in qRT-PCR are listed in **Table 2**.

**Table 2:** List of primers used in qRT-PCR.

Name	Sequence
*ACT1-*F	5′-TTGGATTCCGGTGATGGTGTTACT-3′
*ACT1-*R	5′-TGAAGAAGATTGAGCAGCGGTTTG-3′
*BTN2-*F	5′-TTTCCGAAGGTGGCATC AAC-3′
*BTN2-*R	5′-CTTTCGCTTTCTCCGCTTCTTC-3′
*HSP30-*F	5′-TGGCCTGGATATGCAC ATTA-3′
*HSP30-*R	5′-GACTGCAAACACTGCCCATA-3′
*CUR1-*F	5′-CCTTTCAATGGCAATGGCTTACA-3′
*GIC2-*F	5′-GCTACGCCTTCTCCACAATCTA-3′
*YUR1-*F	5′-ACCTGTCCAGCATCTTACGC-3′
*FLAG-*R	5′-TC ATCGTCATCCTTGTAGTC-3′


### Western Blotting

After the treatment of cells with ethanol stress, cell-free extract (CFE) in 50 mM potassium phosphate buffer (pH 6.8) was prepared. Total protein concentration of the CFE was measured using Protein Assay CBB Solution kit (Nacalai Tesque, Kyoto, Japan). Next, the CFE was mixed with SDS sample buffer solution containing a reducing reagent (6x, Code No. 09499-14; Nacalai Tesque) and was heated at 98°C for 5 min. Thirty six micrograms of total protein was applied to each lane of a 10% polyacrylamide gel for the SDS-PAGE analysis. The resolved proteins were transferred onto polyvinylidene difluoride membranes (Immobilon-P; Merck Millipore Ltd, MA, USA). The blotted membranes were blocked with PBS containing 0.05% Tween 20 and 1% skim milk for 10 min. After washing, the membranes were incubated with anti-FLAG M2 primary antibody (dilution, 1:1,000; Sigma–Aldrich, MO, USA) to monitor the levels of FLAG-tagged proteins (Btn2, Cur1, Gic2, and Yur1). Pgk1 was used as a loading control, and its level was monitored using a monoclonal anti-Pgk1 primary antibody (22C5D8; dilution, 1:4,000; Life Technologies, Frederick, MD, USA). The anti-FLAG and anti-Pgk1 primary antibodies were detected using HRP-conjugated anti-mouse secondary antibody (dilution, 1:1,000; Cell Signaling Technology, Beverly, MA, USA). Antibody binding was detected using Chemi-Lumi One L Western blotting detection reagents (Code No. 07880-70; Nacalai Tesque). The bands of the Western blot were quantified using Image Studio Digits Ver 4.0 software (LI-COR Biotechnology, Lincoln, NE, USA). Levels of FLAG-tagged proteins were normalized to that of Pgk1, and the intensity of the Pgk1 band in each lane was set at 100%.

## Results

### High Concentration Ethanol Stress Caused the Repression of Translation Activity

**FIGURE 1:**
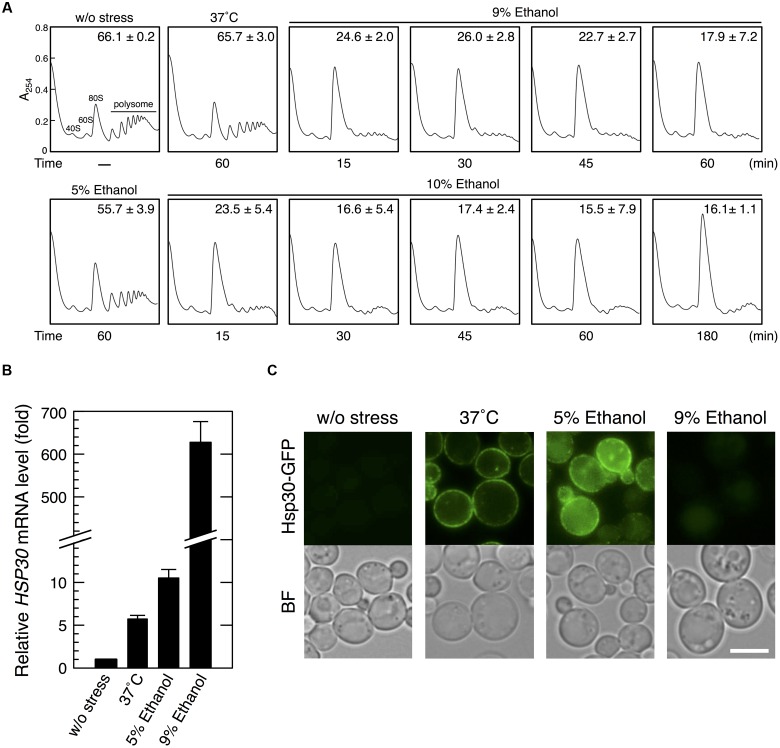
**Severe ethanol stress inhibited translation initiation in yeast cells.** **(A)** The polysome profile of yeast cells under severe ethanol stress was determined. Yeast cells in the exponential growth phase in the SD medium at 28°C were treated with mild heat shock at 37°C for 60 min, 5% ethanol for 60 min, or 9 or 10% (v/v) ethanol (severe ethanol stress) for the indicated time. Polysome, 40S (small ribosomal subunit), 60S (large ribosomal subunit), and 80S (monosome) peaks are labeled. The numbers in the panels indicate the percentages of polysomal ribosomes. Data are expressed as mean ± SD (*n* = 3). **(B,C)** Yeast cells carrying the GFP-tagged chromosomal copy of *HSP30* were treated with 5 or 9% ethanol stress or with mild heat shock at 37°C for 60 min. **(B)** *HSP30* mRNA level was analyzed by performing qRT-PCR and was normalized with that of *ACT1*. Each value is expressed as the mean ± SD of relative fold change in expression normalized to that in untreated control cells (*n* = 3). **(C)** Hsp30-GFP synthesis was detected by performing fluorescence microscopic analysis. Cells under stress were observed immediately after treatment without fixation. Exposure time is the same in all the images. The white bar indicates 5 μm. BF, bright field.

First, we performed polysome profile analysis to verify whether severe ethanol stress repressed translation activity in yeast cells (**Figure 1A**). Treatment of yeast cells with 9 or 10% (v/v) ethanol induced a pronounced rapid reduction in polysome fraction and a concomitant increase in monosome fraction (80S), indicating a significant repression of translation initiation, which was similar to that observed during glucose deprivation (Ashe et al., 2000). Further, prolonged treatment of yeast cells with 10% ethanol maintained ethanol stress-induced translation repression for at least 180 min. These data clearly confirmed that severe ethanol stress (>9% ethanol, v/v) considerably repressed overall protein synthesis in yeast cells. Although mRNA levels of *HSP30* increased in yeast cells treated with 9% ethanol for 60 min (**Figure 1B**), Hsp30-GFP protein expression was negligible as indicated by very weak fluorescence intensity (**Figure 1C**). However, exposure to heat shock at 37°C or treatment with 5% ethanol (mild ethanol stress) induced Hsp30-GFP protein expression in the plasma membrane of yeast cells (**Figure 1C**), indicating that severe ethanol stress repressed Hsp30 protein synthesis. These results confirmed that increased mRNA expression did not necessarily indicate an increase in corresponding protein expression in yeast cells under severe ethanol stress because of the repression of overall protein synthesis.

### *BTN2* Was Preferentially Translated under Severe Ethanol Stress

**FIGURE 2:**
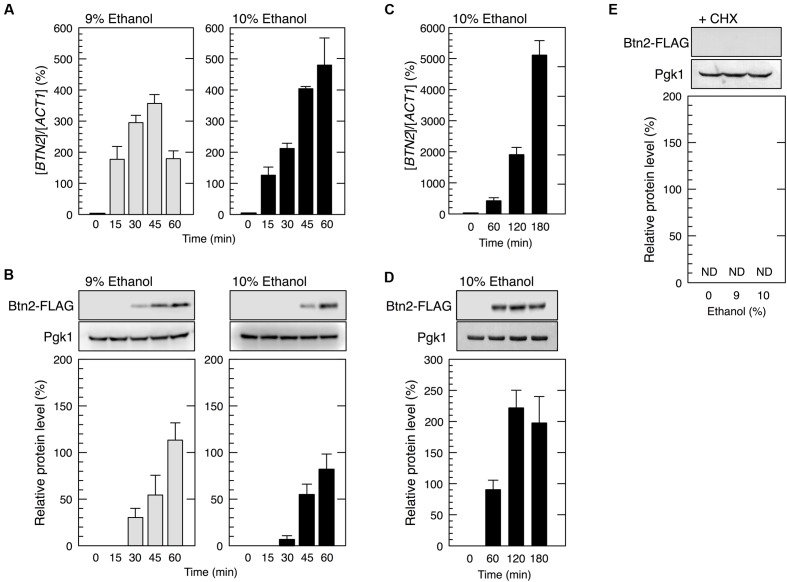
***BTN2* was preferentially expressed under severe ethanol stress.** Yeast cells carrying a FLAG-tagged chromosomal copy of *BTN2* in the exponential growth phase were treated with 9 or 10% (v/v) ethanol (severe ethanol stress) for the indicated time **(A–D)** or treated with 9 or 10% (v/v) ethanol and 0.1 mg/ml cycloheximide (CHX) for 60 min **(E)**. **(A,C)** *BTN2* mRNA level was determined by performing qRT-PCR and was normalized to that of *ACT1*. **(B,D,E)** Btn2 protein level was determined by performing Western blotting with anti-FLAG antibody. Pgk1 was used as the loading control. Btn2 protein level was normalized to that of Pgk1, and the intensity of the Pgk1 band in each lane was set at 100%. Data are expressed as mean ± SD (*n* = 3). ND, not detected.

We next tried to identify genes that were preferentially translated under severe ethanol stress. Since various genes associated with ethanol tolerance have been reported (Alexandre et al., 2001; Takahashi et al., 2001; Fujita et al., 2006; van Voorst et al., 2006; Hirasawa et al., 2007; Teixeira et al., 2009), we randomly examined whether protein levels of those genes were increased by the treatment with 9 or 10% ethanol. As a screening result, only *BTN2* was confirmed as the gene whose protein levels were significantly increased under severe ethanol stress so far. *BTN2* encodes a v-SNARE binding protein and is associated with ethanol tolerance (Chattopadhyay and Pearce, 2002; Kama et al., 2007; Espinazo-Romeu et al., 2008; Yang et al., 2011). We first examined *BTN2* mRNA level in yeast cells under severe ethanol stress (9 or 10% ethanol). Our results showed that *BTN2* mRNA level significantly increased under severe ethanol stress (**Figure 2A**), which was similar to that reported by Cho et al. (2014).

We next examined whether Btn2 protein synthesis was upregulated under severe ethanol stress (**Figure 2B**). Although Btn2 protein expression was negligible in yeast cells under non-stress condition, clear bands representing Btn2 were detected following the treatment with severe ethanol stress. Btn2 protein levels significantly increased within 30 min in cells treated with 9% ethanol and within 45 min in cells treated with 10% ethanol. Prolonged treatment with 10% ethanol further increased Btn2 protein levels (**Figures 2C,D**). These results strongly suggested that *BTN2* expression was induced and that *BTN2* mRNA was efficiently translated in yeast cells under severe ethanol stress, despite the pronounced repression of overall protein synthesis. To verify translation of *BTN2* mRNA under severe ethanol stress, we also investigated protein levels of Btn2 in cells simultaneously treated with severe ethanol stress and cycloheximide (CHX), a strong inhibitor of protein biosynthesis (Obrig et al., 1971). CHX had almost no effect on the transcriptional activation of *BTN2* (data not shown) but completely repressed the increase of Btn2 protein levels (**Figure 2E**) under severe ethanol stress. These results clearly indicate that newly synthesis of Btn2 protein was induced under severe ethanol stress.

**FIGURE 3:**
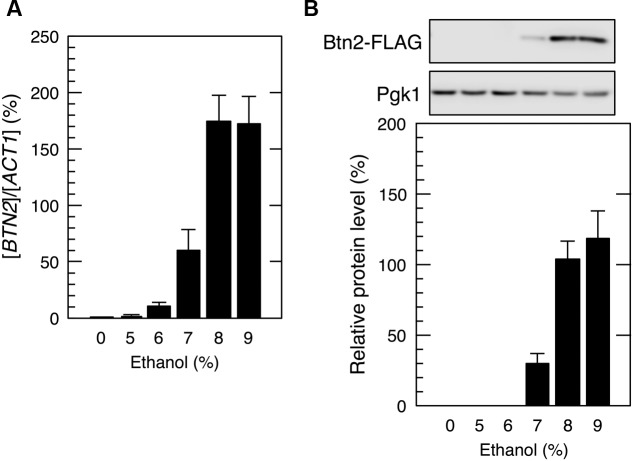
***BTN2* expression under mild ethanol stress.** Cells carrying a FLAG-tagged chromosomal copy of *BTN2* gene in the exponential growth phase were treated with the indicated concentrations of ethanol for 60 min. **(A)** *BTN2* mRNA level was determined by performing qRT-PCR and was normalized to that of *ACT1*. **(B)** Btn2 protein level was determined by performing Western blotting with anti-FLAG antibody. Pgk1 was used as the loading control. Btn2 protein level was normalized to that of Pgk1, and the intensity of the Pgk1 band in each lane was set at 100%. Data are expressed as mean ± SD (*n* = 3).

In contrast, yeast cells exposed to mild ethanol stress (5 and 6% ethanol, v/v) for 60 min did not show the efficient translation of *BTN2* mRNA (**Figure 3**), indicating that *BTN2* was expressed under severe ethanol stress but not under mild ethanol stress.

### Elimination of Ethanol Stress Reconstructed Polysomes and Inhibited Btn2 Protein Expression

**FIGURE 4:**
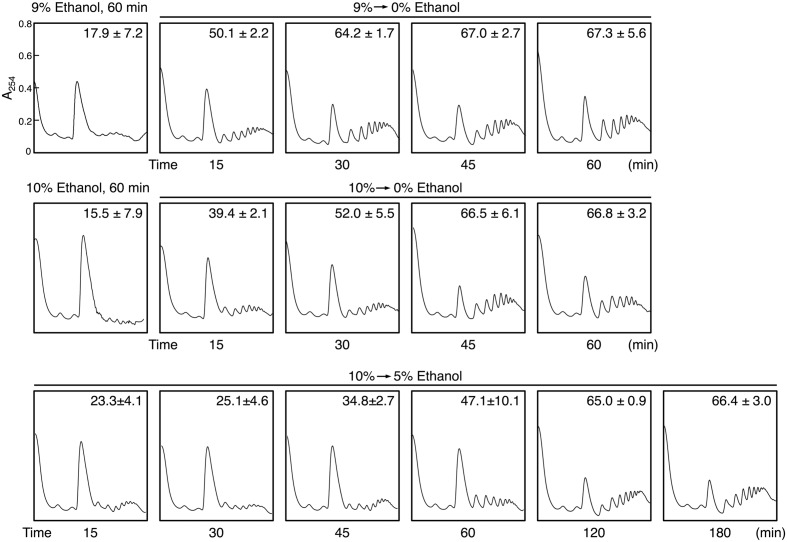
**Reconstruction of polysomes during recovery from severe ethanol stress.** Yeast cells in the exponential growth phase were treated with 9 or 10% (v/v) ethanol (severe ethanol stress) for 60 min. After the treatment with ethanol stress, cells were transferred to a fresh SD medium lacking ethanol or containing 5% ethanol and were incubated at 28°C for the indicated time. The numbers in the panels indicate the percentages of polysomal ribosomes. Data are expressed as mean ± SD (*n* = 3).

**FIGURE 5:**
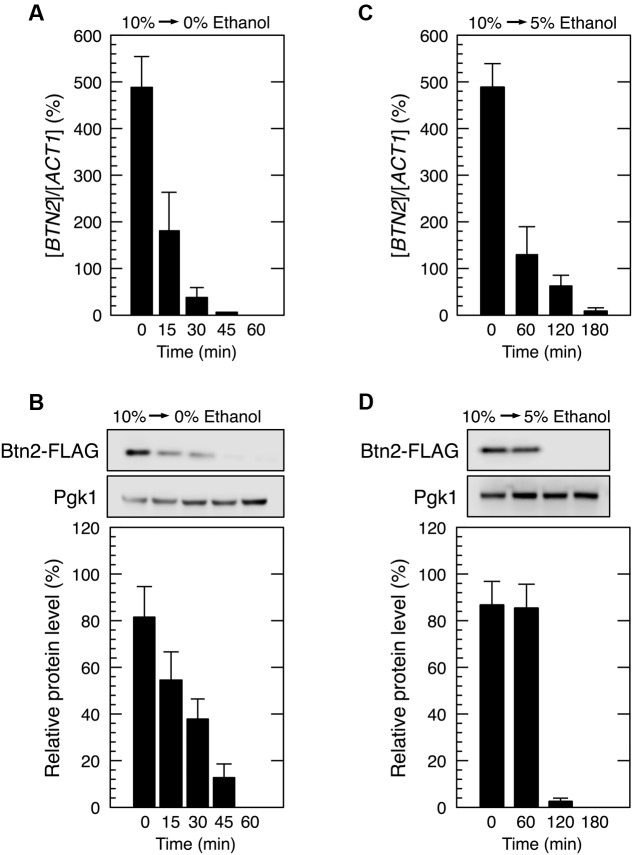
**Btn2 protein level decreased during recovery from severe ethanol stress.** Cells carrying a FLAG-tagged chromosomal copy of *BTN2* gene in the exponential growth phase were treated with severe ethanol stress (10% v/v) for 60 min. After the treatment with ethanol stress, cells were transferred to a fresh SD medium lacking ethanol **(A,B)** or containing 5% ethanol **(C,D)** and were incubated further at 28°C for the indicated time. **(A,C)** *BTN2* mRNA level was determined by performing qRT-PCR and was normalized to that of *ACT1*. **(B,D)** Btn2 protein level was determined by performing Western blotting with anti-FLAG antibody. Pgk1 was used as the loading control. Btn2 protein level was normalized to that of Pgk1, and the intensity of the Pgk1 band in each lane was set at 100%. Data are expressed as mean ± SD (*n* = 3).

We next investigated the restoration of translation activity during recovery from severe ethanol stress (**Figure 4**). Elimination of ethanol by replacing the culture medium (decrease in ethanol concentration from 9 or 10 to 0%) significantly reduced the monosome (80S) fraction and markedly increased the polysome fraction, indicating the restoration of translation activity. Medium replacement efficiently increased the percentage of polysomal ribosomes to the original level within 30–45 min. These results suggested that severe ethanol stress-induced translation repression was smoothly recovered after the elimination of ethanol stress. Next, we examined Btn2 protein levels in yeast cells during the recovery process from severe ethanol stress. Medium replacement not only decreased *BTN2* mRNA levels but also gradually decreased Btn2 protein levels, with negligible Btn2 protein levels being detected within 45 min after medium replacement (**Figures 5A,B**).

However, culturing of 10% ethanol-treated yeast cells in a fresh medium containing 5% ethanol (from 10 to 5% ethanol) did not completely restore the translation activity within 1 h (**Figure 4**). The time required for the reconstruction of polysomes and inhibition of Btn2 protein expression was longer in yeast cells cultured in SD medium containing 5% ethanol than in yeast cells cultured in SD medium lacking ethanol (**Figure 5**).

### The *BTN2* Promoter Region Induces Protein Synthesis under Severe Ethanol Stress

Promoter regions of some genes induce the preferential translation of non-native genes during severe stress-induced translation repression. Zid and O’Shea (2014) reported that the promoters of *HSP26* and *HSP30* increased protein synthesis during glucose deprivation. We also recently reported that the *ADH7* promoter and *BDH2* promoter induced translation under severe vanillin stress, despite the pronounced repression of overall protein synthesis (Nguyen et al., 2015; Ishida et al., 2016). Based on these findings, we investigated whether the *BTN2* promoter induced the protein synthesis of non-native genes under severe ethanol stress.

**FIGURE 6:**
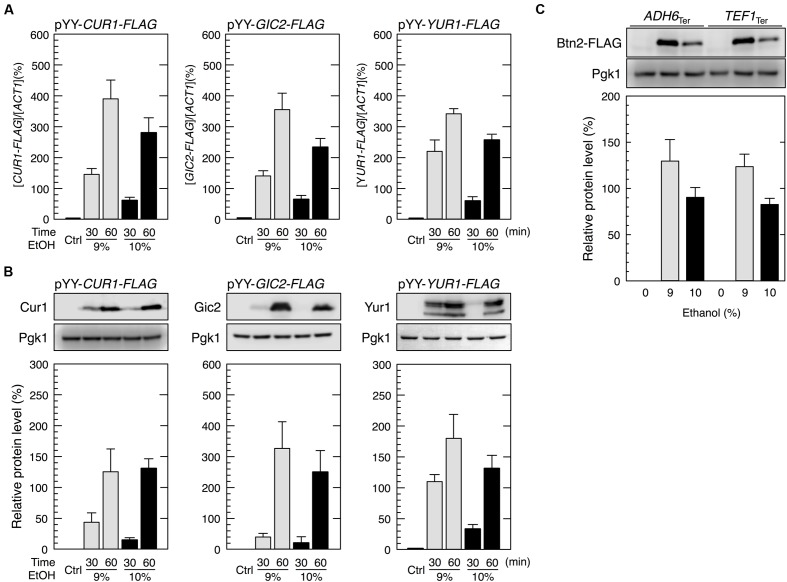
**The *BTN2* promoter region induced the protein synthesis of non-native genes under severe ethanol stress.** **(A,B)** Cells harboring the pYY plasmid series (*BTN2* promoter-driven expression system) in the exponential growth phase were treated with 9 or 10% ethanol for the indicated time. **(A)** The mRNA levels of each FLAG-tagged gene were analyzed by performing qRT-PCR and were normalized to that of *ACT1*. **(B)** Protein levels of Cur1-FLAG, Gic2-FLAG, and Yur1-FLAG were determined by performing Western blotting with anti-FLAG antibody. **(C)** The *BTN2* terminator region had a negligible effect on preferential translation upon severe ethanol stress. Cells carrying YIp-*BTN2-FLAG-ADH6*_Ter_ or YIp-*BTN2-FLAG-TEF1*_Ter_ in the exponential phase of growth were treated with 9 or 10% ethanol for 1 h. Protein levels of Btn2-FLAG were determined by Western blot analysis using an anti-FLAG antibody. Pgk1 was used as the loading control. Their protein levels were normalized to that of Pgk1, and the intensity of the Pgk1 band in each lane was set at 100%. Data are shown as mean ± SD (*n* = 3). Ctrl, control cells not treated with ethanol stress.

We constructed an expression system containing the promoter and terminator regions of *BTN2* (pYY2712, see Materials and Methods) flanking the ORFs of target genes. We examined three genes, *CUR1*, *GIC2*, and *YUR1*. *CUR1* was chosen as a paralog of *BTN2* (Byrne and Wolfe, 2005; Malinovska et al., 2012). *GIC2* and *YUR1* were randomly chosen from yeast genes. Yeast cells harboring pYY*-CUR1-FLAG* showed increased protein levels of Cur1, a sorting factor and central regulator of spatial protein quality control (Kryndushkin et al., 2008), under severe ethanol stress (**Figure 6**). Similarly, yeast cells carrying pYY*-GIC2-FLAG* or pYY*-YUR1-FLAG* showed increased protein levels of Gic2, a rho-like GTPase Cdc42 effector (Chen et al., 1997), or Yur1, a mannosyltransferase (Lussier et al., 1997), under severe ethanol stress. Since replacement of the *BTN2* terminator region with other terminator regions (*ADH6* terminator region and *TEF1* terminator region) did not affect the induction of Btn2 protein synthesis (**Figure 6C**), contribution of the *BTN2* terminator to the preferential translation of *BTN2* under severe ethanol stress seems negligible. These results strongly suggested that the *BTN2* promoter region induced the expression of its regulated genes under severe ethanol stress.

## Discussion

In the present study, we performed polysome profile analysis to verify that high ethanol concentration severely repressed the bulk translation activity in yeast cells. The pronounced translation repression was maintained for at least 180 min after exposure to 10% ethanol stress (**Figure 1A**). Compared with high salinity stress (1 M NaCl), which induces pronounced but transient translation repression (Uesono and Toh-E, 2002; Melamed et al., 2008), severe ethanol stress induced long-term translation repression. However, elimination of ethanol stress rapidly restored translation activity (**Figure 4**), clearly indicating that severe ethanol stress did not irreparably damage the components of the translation apparatus.

Next, we tried to identify mRNAs that overcome severe ethanol stress-induced translation repression and found that *BTN2* mRNA was preferentially translated despite the pronounced repression of overall protein synthesis (**Figure 2**). The null mutant of *BTN2* is hypersensitive to ethanol (Espinazo-Romeu et al., 2008; Yang et al., 2011), suggesting that *BTN2* expression through its preferential translation is necessary for inducing resistance to severe ethanol stress. However, severe ethanol stress-induced Btn2 protein expression was abolished after eliminating ethanol stress (**Figure 5**). It has been reported that Btn2 protein levels were low and detection of Btn2 was relatively difficult under physiological growth conditions (Chattopadhyay and Pearce, 2002; Kryndushkin et al., 2008; Malinovska et al., 2012; Miller et al., 2015). Consistently, we also observed that Btn2 protein levels were very low under mild ethanol stress (**Figure 3**). Additionally, overexpression of *BTN2* causes the delay of cell growth (Sopko et al., 2006). These previous reports and our results suggested that *BTN2* is expressed under severe ethanol stress but not under mild ethanol stress and that *BTN2* expression is strictly repressed under non-severe ethanol stress conditions.

Heat shock elements in the promoter regions are crucial for inducing preferential translation under glucose deprivation (Zid and O’Shea, 2014). The *BTN2* promoter region contains three HSEs, namely, HSE1, 5′-AGAAAGTTCCGGAAA-3′ (from -340 to -326 relative to the translation initiation ATG codon); HSE2, 5′-GGAAGAATTAAGAATTTCATAGAAG-3′ (from -230 to -206); and HSE3, 5′-ATGGAAGA-3′ (from -170 to -163) (Slater and Craig, 1987; Yamamoto et al., 2005; Badis et al., 2008). These HSEs are crucial for the transcriptional activation of *BTN2* under severe ethanol stress (Cho et al., 2014). Additionally, they may play a role in the prioritized translation of *BTN2* under severe ethanol stress as well as glucose deprivation. A recent study reported that mammalian translation elongation factor eEF1A is recruited to HSEs in the *HSP70* promoter region to promote Hsp70 protein synthesis by coupling transcription with translation during heat shock-induced translation repression (Vera et al., 2014). Similarly, some factor(s) that promote efficient translation under severe stress might be recruited to HSEs in yeast cells. However, presence of HSEs in promoter regions may not be sufficient for inducing effective translation under severe ethanol stress, unlike that observed during glucose deprivation, because translation of HSE-containing genes such as *HSP30* and *HSP82* is negligible under severe ethanol stress despite of their transcription (**Figure 1**) (Izawa et al., 2008). Mechanisms underlying preferential translation under severe ethanol stress might be different from those underlying preferential translation under glucose deprivation. Identification of essential *cis-* and *trans-*elements for preferential translation under severe ethanol stress is in progress. Identification of the essential *cis-*element(s) in the *BTN2* promoter regions will facilitate the identification of other genes that are preferentially translated under severe ethanol stress.

We successfully expressed genes other than *BTN2* under severe ethanol stress by using the *BTN2* promoter region (**Figure 6**). The ability of the *BTN2* promoter to overcome translation repression induced by severe ethanol stress could be very useful for improving the fermentation ability of yeast cells. For example, the *BTN2* promoter could be used to express genes important for ethanol production, which are usually repressed under ethanol stress, for increasing and stabilizing the fermentation activity of yeast cells. The *BTN2* promoter could also be used to alter the quality of wine and *sake* by modifying gene expression pattern in the final stage of brewing. Furthermore, utilization of the *BTN2* promoter might realize efficient parallel-production of ethanol and useful proteins such as vaccines and hormones during wine making and Japanese *sake* brewing.

## Author Contributions

YY did most of experiments and SI did several experiments and mainly prepared the manuscript.

## Conflict of Interest Statement

The authors declare that the research was conducted in the absence of any commercial or financial relationships that could be construed as a potential conflict of interest.

## References

[B1] Alexandre H. Ansanay-Galeote V. Dequin S. Blondin B. (2001). Global gene expression durig short-term ethanol stress in *Saccharomyces cerevisiae*. *FEBS Lett.* 498 98–103. 10.1016/S0014-5793(01)02503-0 11389906

[B2] Ashe M. P. De Long S. K. Sachs A. B. (2000). Glucose depletion rapidly inhibits translation initiation in yeast. *Mol. Biol. Cell* 11 833–848. 10.1091/mbc.11.3.833 10712503 PMC14814

[B3] Badis G. Chan E. T. van Bakel H. Pena-Castillo L. Tillo D. Tsui K. (2008). A library of yeast transcription factor motifs reveals a widespread function for Rsc3 in targeting nucleosome exclusion at promoters. *Mol. Cell* 32 878–887. 10.1016/j.molcel.2008.11.020 19111667 PMC2743730

[B4] Balagopal V. Parker R. (2009). Polysomes, P bodies and stress granules: states and fates of eukaryotic mRNAs. *Curr. Opin. Cell Biol.* 21 403–408. 10.1016/j.ceb.2009.03.005 19394210 PMC2740377

[B5] Buchan J. R. Parker R. (2009). Eukaryotic stress granules: the ins and outs of translation. *Mol. Cell* 36 932–940. 10.1016/j.molcel.2009.11.020 20064460 PMC2813218

[B6] Buchan J. R. Yoon J. H. Parker R. (2011). Stress-specific composition, assembly and kinetics of stress granules in *Saccharomyces cerevisiae*. *J. Cell Sci.* 124 228–239. 10.1242/jcs.078444 21172806 PMC3010191

[B7] Byrne K. P. Wolfe K. H. (2005). The yeast gene order browser: combining curated homology and syntenic context reveals gene fate in polyploid species. *Genome Res.* 15 1456–1461. 10.1101/gr.3672305 16169922 PMC1240090

[B8] Chattopadhyay S. Muzaffar N. E. Sherman F. Pearce D. A. (2000). The yeast model for Batten disease: mutations in btn1, btn2, and hsp30 alter pH homeostasis. *J. Bacteriol.* 182 6418–6423. 10.1128/JB.182.22.6418-6423.2000 11053386 PMC94788

[B9] Chattopadhyay S. Pearce D. A. (2002). Interaction with Btn2p is required for localization of Rsglp: Btn2p-mediated changes in arginine uptake in *Saccharomyces cerevisiae*. *Eukaryot. Cell* 1 606–612. 10.1128/EC.1.4.606-612.2002 12456008 PMC117998

[B10] Chen G. C. Kim Y. J. Chen C. S. (1997). The Cdc42 GTPase-associated proteins Gic1 and Gic2 are required for polarized cell growth in *Saccharomyces cerevisiae*. *Genes Dev.* 11 2958–2971. 10.1101/gad.11.22.2958 9367979 PMC316704

[B11] Cho B. Lee P. Hahn J. (2014). CK2-dependent inhibitory phosphorylation is relieved by Ppt1 phosphatase for the ethanol stress-specific activation of Hsf1 in *Saccharomyces cevervisiae*. *Mol. Microbiol.* 93 306–316. 10.1111/mmi.12660 24894977

[B12] Espinazo-Romeu M. Cantoral J. M. Matallana E. Aranda A. (2008). Btn2p is involved in ethanol tolerance and biofilm formation in flor yeast. *FEMS Yeast Res.* 8 1127–1136. 10.1111/j.1567-1364.2008.00397.x 18554307

[B13] Fujita K. Matsuyama A. Kobayashi Y. Iwahashi H. (2006). The genome-wide screening of yeast deletion mutants to identify the genes required for tolerance to ethanol and other alcohols. *FEMS Yeast Res.* 6 744–750. 10.1111/j.1567-1364.2006.00040.x 16879425

[B14] Goto K. Nagano Y. (2013). Ultra-low background DNA cloning system. *PLoS ONE* 8:e56530 10.1371/journal.pone.0056530 PMC3568078 23409191

[B15] Grousl T. Ivanov P. Frydlova I. Vasicova P. Janda F. Vojtova J. (2009). Robust heat shock induces eIF2-phosphorylation-independent assembly of stress granules containing eIF3 and 40S ribosomal subunits in budding yeast, *Saccharomyces cerevisiae*. *J. Cell Sci.* 122 2078–2088. 10.1242/jcs.045104 19470581

[B16] Hirasawa T. Yoshikawa K. Nakakura Y. Nagahisa K. Furusawa C. Katakura Y. (2007). Identification of target genes conferring ethanol stress tolerance to *Saccharomyces cerevisiae* based on DNA microarray data analysis. *J. Biotechnol.* 131 34–44. 10.1016/j.jbiotec.2007.05.010 17604866

[B17] Hofmann S. Cherkasova V. Bankhead P. Bukau B. Stoecklin G. (2012). Translation suppression promotes stress granule formation and cell survival in response to cold shock. *Mol. Biol. Cell* 23 3786–3800. 10.1091/mbc.E12-04-0296 22875991 PMC3459856

[B18] Inada T. Aiba H. (2005). Translation of aberrant mRNAs lacking a termination codon or with a shortened 3′-UTR is repressed after initiation in yeast. *EMBO J.* 24 1584–1595. 10.1038/sj.emboj.7600636 15933721 PMC1142571

[B19] Ishida Y. Nguyen T. T. M. Kitajima S. Izawa S. (2016). Prioritized expression of *BDH2* under bulk translational repression and its contribution to tolerance to severe vanillin stress in *Saccharomyces cerevisiae*. *Front. Microbiol.* 7:1059 10.3389/fmicb.2016.01059 PMC4933698 27458450

[B20] Iwaki A. Kawai T. Yamamoto Y. Izawa S. (2013). Biomass conversion inhibitors, furfural and 5-hydroxymethylfurfural, induce the formation of mRNP granules and attenuate translation activity in yeast. *Appl. Environ. Microbiol.* 446 225–233. 10.1128/AEM.02797-12 PMC3591974 23275506

[B21] Izawa S. (2010). Ethanol stress response in mRNA flux of *Saccharomyces cerevisiae*. *Biosci. Biotechnol. Biochem.* 74 7–12. 10.1271/bbb.90686 20057118

[B22] Izawa S. (2015). “Yeast mRNA flux during brewing and under ethanol stress conditions,” in *Stress Biology of Yeast and Fungi*, eds Takagi H. Kitagaki H. (Tokyo: Springer), 43–57.

[B23] Izawa S. Kita T. Ikeda K. Inoue Y. (2008). Heat shock and ethanol stress provoke distinctly different responses in 3′-processing and nuclear export of HSP mRNA in *Saccharomyces cerevisiae*. *Biochem. J.* 414 111–119. 10.1042/BJ20071567 18442359

[B24] Izawa S. Kita T. Ikeda K. Miki T. Inoue Y. (2007). Formation of the cytoplasmic P-bodies in sake yeast during Japanese sake brewing and wine making. *Biosci. Biotechnol. Biochem.* 71 2800–2807. 10.1271/bbb.70417 17986786

[B25] Izawa S. Takemura R. Ikeda K. Fukuda K. Wakai Y. Inoue Y. (2005a). Characterization of Rat8 localization and mRNA export in *Saccharomyces cerevisiae* during the brewing of Japanese sake. *Appl. Microbiol. Biotechnol.* 69 86–91. 10.1007/s00253-005-1954-x 15803312

[B26] Izawa S. Takemura R. Miki T. Inoue Y. (2005b). Characterization of the export of bulk poly(A)^+^ mRNA in *Saccharomyces cerevisiae* during the wine-making process. *Appl. Environ. Microbiol.* 71 2179–2182. 10.1128/AEM.71.4.2179-2182.2005 15812055 PMC1082520

[B27] Kahana J. A. Schlenstedt G. Evanchuk D. M. Geiser J. R. Hoyt M. A. Silver P. A. (1998). The yeast dynactin complex is involved in partitioning the mitotic spindle between mother and daughter cells during anaphase B. *Mol. Biol. Cell* 9 1741–1756. 10.1091/mbc.9.7.1741 9658168 PMC25412

[B28] Kama R. Robinson M. Gerst J. E. (2007). Btn2, a hook1 ortholog and potential batten disease-related protein, mediates late endosome-Golgi protein sorting in yeast. *Mol. Cell. Biol.* 27 605–621. 10.1128/MCB.00699-06 17101785 PMC1800815

[B29] Kato K. Yamamoto Y. Izawa S. (2011). Severe ethanol stress induces assembly of stress granules in *Saccharomyces cerevisiae*. *Yeast* 28 339–347. 10.1002/yea.1842 21341306

[B30] Kim Y. Chattopadhyay S. Locke S. Pearce D. A. (2005). Interaction among Btn1p, Btn2p, and Ist2p reveals potential interplay among the vacuole, amino acid levels, and ion homeostasis in the yeast *Saccharomyces cerevisiae*. *Eukaryot. Cell* 4 281–288. 10.1128/EC.4.2.281-288.2005 15701790 PMC549324

[B31] Kryndushkin D. S. Shewmaker F. Wickner R. B. (2008). Curing of the [URE3] prion by Btn2p, a Batten disease-related protein. *EMBO J.* 27 2725–2735. 10.1038/emboj.2008.198 18833194 PMC2572181

[B32] Lussier M. Sdicu A. M. Camirand A. Bussey H. (1997). Functional characterization of the *YUR1*, *KTR1*, and *KTR2* genes as members of the yeast *KRE2/MNT1* mannosyltransferase gene family. *J. Biol. Chem.* 271 11001–11008. 10.1074/jbc.271.18.11001 8631921

[B33] Malinovska L. Kroschwald S. Munder M. C. Richter D. Alberti S. (2012). Molecular chaperones and stress-inducible protein-sorting factors coordinate the spatiotemporal distribution of protein aggregates. *Mol. Biol. Cell* 23 3041–3056. 10.1091/mbc.E12-03-0194 22718905 PMC3418301

[B34] Melamed D. Pnueli L. Arava Y. (2008). Yeast translational response to high salinity: global analysis reveals regulation at multiple levels. *RNA* 14 1337–1351. 10.1261/rna.864908 18495938 PMC2441982

[B35] Miller S. B. M. Ho C. Winkler J. Khokhrina M. Neuner A. Mohamed M. Y. H. (2015). Compartment-specific aggregases direct distinct nuclear and cytoplasmic aggregate deposition. *EMBO J.* 34 778–797. 10.15252/embj.201489524 25672362 PMC4369314

[B36] Nguyen T. T. M. Iwaki A. Izawa S. (2015). The *ADH7* promoter of *Saccharomyces cerevisiae* is vanillin-inducible and enables mRNA translation under severe vanillin stress. *Front. Microbiol.* 6:1390 10.3389/fmicb.2015.01390 PMC4676198 26696995

[B37] Nguyen T. T. M. Kitajima S. Izawa S. (2014). Importance of glucose-6-phosphate dehydrogenase (G6PDH) for vanillin tolerance in *Saccharomyces cerevisiae*. *J. Biosci. Bioeng.* 118 263–269. 10.1016/j.jbiosc.2014.02.025 24725964

[B38] Obrig T. G. Culp W. J. McKeehan W. L. Hardesty B. (1971). The mechanism by which cycloheximide and related glutarimide antibiotics inhibit peptide synthesis on reticulocyte ribosomes. *J. Biol. Chem.* 246 174–181. 5541758

[B39] Schmitt M. E. Brown T. A. Trumpower B. L. (1990). A rapid and simple method for preparation of RNA from *Saccharomyces cerevisiae*. *Nucleic Acids Res.* 18 3091–3092. 10.1093/nar/18.10.3091 2190191 PMC330876

[B40] Sikorski R. S. Hieter P. (1989). A system of shuttle vectors and yeast host strains designed for efficient manipulation of DNA in *Saccharomyces cerevisiae*. *Genetics* 122 19–27. 2659436 10.1093/genetics/122.1.19 PMC1203683

[B41] Slater M. R. Craig E. A. (1987). Transcriptional regulation of an hsp70 heat shock gene in the yeast *Saccharomyces cerevisiae*. *Mol. Cell. Biol.* 7 1906–1916. 10.1128/MCB.7.5.1906 3037338 PMC365295

[B42] Sopko R. Huang D. Preston N. Chua G. Papp B. Kafadar K. (2006). Mapping pathways and phenotypes by systematic gene overexpression. *Mol. Cell* 21 319–330. 10.1016/j.molcel.2005.12.011 16455487

[B43] Takahashi T. Satake S. Hirose K. Hwang G. H. Naganuma A. (2011). A screening for essential cell growth-related genes involved in arsenite toxicity in *Saccharomyces cerevisiae*. *J. Toxicol. Sci.* 36 859–861. 10.2131/jts.36.859 22129753

[B44] Takahashi T. Shimoi H. Ito K. (2001). Identification of genes required for growth under ethanol stress using transposon mutagenesis in *Saccharomyces cerevisiae*. *Mol. Genet. Genomics* 265 1112–1119. 10.1007/s004380100510 11523784

[B45] Takemura R. Inoue Y. Izawa S. (2004). Stress response in yeast mRNA export factor: reversible changes in Rat8p localization are caused by ethanol stress but not heat shock. *J. Cell Sci.* 117 4189–4197. 10.1242/jcs.01296 15280434

[B46] Teixeira D. Sheth U. Valencia-Sanchez M. A. Brengues M. Parker R. (2005). Processing bodies require RNA for assembly and contain nontranslating mRNAs. *RNA* 11 371–382. 10.1261/rna.7258505 15703442 PMC1370727

[B47] Teixeira M. C. Raposo L. R. Mira N. P. Lourenço A. B. Sá-Correia I. (2009). Genome-wide identification of *Saccharomyces cerevisiae* genes required for maximal tolerance to ethanol. *Appl. Environ. Microbiol.* 75 5761–5772. 10.1128/AEM.00845-09 19633105 PMC2747848

[B48] Uesono Y. Toh-E A. (2002). Transient inhibition of translation initiation by osmotic stress. *J. Biol. Chem.* 277 13848–13855. 10.1074/jbc.M108848200 11796711

[B49] van Voorst F. Houghton-Larsen J. Jønson L. Kielland-Brandt M. C. Brandt A. (2006). Genome-wide identification of genes required for growth of *Saccharomyces cerevisiae* under ethanol stress. *Yeast* 23 351–359. 10.1002/yea.1359 16598687

[B50] Vera M. Pani B. Griffiths L. A. Muchardt C. Abbott C. M. Singer R. H. (2014). The translation elongation factor eEF1A1 couples transcription to translation during heat shock response. *eLife* 3:e03164 10.75554/eLife.03164 PMC4164936 25233275

[B51] Yamamoto A. Mizukami Y. Sakurai H. (2005). Identification of a novel class of target genes and a novel type of binding sequence of heat shock transcription factor in *Saccharomyces cerevisiae*. *J. Biol. Chem.* 280 11911–11919. 10.1074/jbc.M411256200 15647283

[B52] Yamamoto Y. Izawa S. (2013). Adaptive response in stress granule formation and bulk translational repression upon a combined stress of mild heat shock and mild ethanol stress in yeast. *Genes Cells* 18 974–984. 10.1111/gtc.12090 24033457

[B53] Yang J. Bae J. Y. Lee Y. M. Kwon H. Moon H. Y. Kang H. A. (2011). Construction of *Saccharomyces cerevisiae* strains with enhanced ethanol tolerance by mutagenesis of the TATA-binding protein gene and identification of novel genes associated with ethanol tolerance. *Biotechnol. Bioeng.* 108 1776–1787. 10.1002/bit.23141 21437883

[B54] Zid B. M. O’Shea E. K. (2014). Promoter sequences direct cytoplasmic localization and translation of mRNAs during starvation in yeast. *Nature* 514 117–121. 10.1038/nature13578 25119046 PMC4184922

